# Profiling of Microbiota at the Mouth of Bottles and in Remaining Tea after Drinking Directly from Plastic Bottles of Tea

**DOI:** 10.3390/dj9060058

**Published:** 2021-05-21

**Authors:** Anna Wakui, Hiroto Sano, Yuka Hirabuki, Miho Kawachi, Ayaka Aida, Jumpei Washio, Yuki Abiko, Gen Mayanagi, Keiko Yamaki, Kaori Tanaka, Nobuhiro Takahashi, Takuichi Sato

**Affiliations:** 1 Division of Clinical Chemistry, Niigata University Graduate School of Health Sciences, Niigata 951-8518, Japan; b20c502h@mail.cc.niigata-u.ac.jp (A.W.); b20c501k@mail.cc.niigata-u.ac.jp (H.S.); yuka.hirabuki1214@gmail.com (Y.H.); b21m202h@mail.cc.niigata-u.ac.jp (M.K.); aayaka0211@gmail.com (A.A.); 2 Division of Oral Ecology and Biochemistry, Tohoku University Graduate School of Dentistry, Sendai 980-8575, Japan; jumpei.washio.b7@tohoku.ac.jp (J.W.); yuki.abiko.a8@tohoku.ac.jp (Y.A.); genm@dent.tohoku.ac.jp (G.M.); k-yamaki@dent.tohoku.ac.jp (K.Y.); nobuhiro.takahashi.a5@tohoku.ac.jp (N.T.); 3 Division of Anaerobic Research, Life Science Research Center, Gifu University, Gifu 501-1194, Japan; kktb@gifu-u.ac.jp

**Keywords:** oral microbiota, PET bottle, profiling, unsweetened tea

## Abstract

It has been speculated that oral bacteria can be transferred to tea in plastic bottles when it is drunk directly from the bottles, and that the bacteria can then multiply in the bottles. The transfer of oral bacteria to the mouth of bottles and bacterial survival in the remaining tea after drinking directly from bottles were examined immediately after drinking and after storage at 37 °C for 24 h. Twelve healthy subjects (19 to 23 years of age) were asked to drink approximately 50 mL of unsweetened tea from a plastic bottle. The mouths of the bottles were swabbed with sterile cotton, and the swabs and the remaining tea in the bottles were analyzed by anaerobic culture and 16S rRNA gene sequencing. Metagenomic analysis of the 16S rRNA gene was also performed. The mean amounts of bacteria were (1.8 ± 1.7) × 10^4^ colony-forming units (CFU)/mL and (1.4 ± 1.5) × 10^4^ CFU/mL at the mouth of the bottles immediately after and 24 h after drinking, respectively. In contrast, (0.8 ± 1.6) × 10^4^ CFU/mL and (2.5 ± 2.6) × 10^6^ CFU/mL were recovered from the remaining tea immediately after and 24 h after drinking, respectively. *Streptococcus* (59.9%) were predominant at the mouth of the bottles immediately after drinking, followed by *Schaalia* (5.5%), *Gemella* (5.5%), *Actinomyces* (4.9%), *Cutibacterium* (4.9%), and *Veillonella* (3.6%); the culture and metagenomic analyses showed similar findings for the major species of detected bacteria, including *Streptococcus* (59.9%, and 10.711%)*, Neisseria* (1.6%, and 24.245%)*, Haemophilus* (0.6%, and 15.658%)*, Gemella* (5.5%, and 0.381%)*, Cutibacterium* (4.9%, and 0.041%)*, Rothia* (2.6%, and 4.170%)*, Veillonella* (3.6%, and 1.130%)*, Actinomyces* (4.9%, and 0.406%)*, Prevotella* (1.6%, and 0.442%)*, Fusobacterium* (1.0%, and 0.461%)*, Capnocytophaga* (0.3%, and 0.028%), and *Porphyromonas* (1.0%, and 0.060%), respectively. Furthermore, *Streptococcus* were the most commonly detected bacteria 24 h after drinking. These findings demonstrated that oral bacteria were present at the mouth of the bottles and in the remaining tea after drinking.

## 1. Introduction

Japanese people often drink tea in polyethylene terephthalate (PET) bottles to stay hydrated. It has been speculated that oral bacteria can be transferred to the tea when it is drunk directly from bottles, and that the bacteria can then multiply in the bottles. In general, it is recommended that any remaining tea in plastic bottles should be discarded after drinking [1,2]; thus, there is currently limited scientific information on the concentration and composition of bacteria in the remaining tea in plastic bottles after drinking directly from the bottles. However, some consumers may be inclined to store or leave the remaining tea in plastic bottles after drinking and then drink it later, especially in the summertime or in hot weather to prevent dehydration. In fact, it has been reported that some bacteria can remain in plastic bottles after drinking [1,2]; however, most of these studies focused mainly on general (water resource-related) bacteria, such as *Escherichia coli*, and the detailed profiles of microbes in the bottles of remaining tea have not yet been clarified.

In this study, in order to understand the characteristics of the bacteria in the remaining tea in plastic bottles after drinking directly from the bottles, analyses of the bacteria at the mouth of bottles and in the remaining tea in the bottles both immediately after drinking from the bottles and after storage at 37 °C for 24 h were performed under culture conditions suitable for the growth of oral microbiota, since the obtained bacteria are assumed to originate from the oral cavity. In addition, the obtained data were confirmed by metagenomic analysis of the 16S rRNA gene.

## 2. Materials and Methods

### 2.1. Sampling

Twelve healthy subjects (19 to 23 years of age) were asked to drink approximately 50 mL of unsweetened tea from a PET bottle (Sokenbicha^®^; Coca-Cola (Japan) Company, Ltd., Tokyo, Japan). All subjects were considered healthy based on their medical history, and none had received antibiotics during the three months before sampling.

Both immediately after drinking and after storage at 37 °C for 24 h, the mouth of each bottle was wiped with a sterile cotton swab. The swab was suspended in 1.0 mL of sterilized 40 mM potassium phosphate buffer (pH 7.0) and dispersed by vortexing. Samples (1.0 mL each) of the remaining tea were also dispersed by vortexing. Serial 10-fold dilutions (0.1 mL each) of the samples in sterilized buffer were spread on the surfaces of CDC Anaerobe 5% Sheep Blood Agar plates (BD, Franklin Lakes, NJ, USA), and incubated anaerobically at 37 °C. After incubation of seven days, the colony-forming units (CFU) were determined, and all colonies from suitably diluted plates having <100 colonies (mean, 21.1 colonies; range, 4 to 38 colonies) were sub-cultured. Because the CFU was higher after anaerobic (approximately 10^4^ CFU/mL) than after aerobic incubation (approximately 10^3^ CFU/mL) at the mouth of the plastic bottles and in the remaining tea immediately after drinking, colonies for further inspections were selected only from plates incubated anaerobically.

### 2.2. pH Measurement

The pH of the tea was measured using a pH meter (HM-25R; DKK-TOA Corporation, Tokyo, Japan).

### 2.3. Identification of Isolates by DNA Sequence Analysis

Genomic DNA was extracted from single colonies using an InstaGene Matrix Kit (Bio-Rad Laboratories, Richmond, CA, USA), and the 16S rRNA gene sequences were amplified by PCR using universal primers, 27F and 1492R, and *Taq* DNA polymerase (HotStar*Taq* Plus Master Mix Kit; Qiagen GmbH, Hilden, Germany), as described before [3,4]. The PCR products were separated on 2% agarose gels (High Strength Analytical Grade Agarose; Bio-Rad Laboratories) as described before [3,4].

Isolates were tentatively identified according to PCR-restriction fragment length polymorphism analysis [3,4]. Then, representative isolates were conclusively identified by sequence analysis, as described before [3,4]. 

### 2.4. Metagenomic Analysis

Genomic DNA was extracted from the swab samples that were suspended in 40 mM potassium phosphate buffer (as described above) using a Mora-Extract Kit (Cosmo Bio) according to the manufacturer’s instructions. 16S ribosomal DNA amplification, library construction, and Illumina MiSeq sequencing were performed at the Oral Microbiome Center (Takamatsu, Japan) using a MiSeq Reagent Kit (Illumina, San Diego, CA, USA), and the Illumina MiSeq platform (Illumina) according to the 16S Metagenomics Sequencing Library Preparation guide supplied by Illumina. The primer sequences were as follows: 341F, 5′- TCG TCG GCA GCG TCA GAT GTG TAT AAG AGA CAG CCT ACG GGN GGC WGC AG -3′, and 806R, 5′- GTC TCG TGG GCT CGG AGA TGT GTA TAA GAG ACA GGG ACT ACH VGG GTW TCT AAT -3′. Microbiome analysis was performed using Qiime (ver. 1.9.1, http://qiime.org/, accessed on 24 April 2021), and the V3–V4 region of the 16S rRNA sequences in the human oral microbiome database (ver. 14.51, http://www.homd.org/, accessed on 24 April 2021).

### 2.5. Statistical Analysis

Dunn’s test was used to determine the statistical significance differences using StatFlex statistical software (ver. 6; Artech Co., Ltd., Osaka, Japan). *p*-values of <0.05 were considered statistically significant.

## 3. Results and Discussion

The mean amounts of bacteria were (1.8 ± 1.7) × 10^4^ CFU/mL and (1.4 ± 1.5) × 10^4^ CFU/mL at the mouth of the plastic bottles immediately after drinking and one day after drinking, respectively (Table 1). In contrast, (0.8 ± 1.6) × 10^4^ CFU/mL and (2.5 ± 2.6) × 10^6^ CFU/mL were recovered from the remaining tea immediately after drinking and 24 h after drinking, respectively (Table 1); the levels immediately after drinking and 24 h after drinking were significantly different (*p* < 0.05). The bacterial levels increased 100-fold (from 10^4^ to 10^6^) after storage at 37 °C for 24 h; this was likely due to the neutral pH (range, pH 5.77 to 6.08) of the tea in the present study, and indicated that the remaining drinks (such as tea drinks) should be preserved in a refrigerator from the viewpoint of bacterial growth. In a preliminary study, no drastic increase in the bacterial amounts, (4.2 ± 5.2) × 10^3^ and (2.5 ± 3.7) × 10^4^ CFU/mL, was observed in tea after storage at 4 °C and 24 °C, respectively, and also, no bacteria were detected in the tea in plastic bottles before drinking.

From the cultures (Table 1), *Streptococcus* species (59.9%) were predominant at the mouth of bottles immediately after drinking, followed by *Schaalia* (5.5%), *Gemella* (5.5%), *Actinomyces* (4.9%), *Cutibacterium* (4.9%), and *Veillonella* (3.6%). From the metagenomic analysis (Table 2), *Delftia* (34.506%), *Neisseria* (24.245%), *Haemophilus* (15.658%), *Streptococcus* (10.711%), *Rothia* (4.170%), *Lautropia* (3.083%), *Sphingomonas* (1.745%), *Ralstonia* (1.436%), and *Veillonella* (1.130%) were predominant, followed by *Fusobacterium* (0.461%), *Prevotella* (0.442%), *Actinomyces* (0.406%), *Agrobacterium* (0.394%), and *Gemella* (0.381%). The culture and metagenomic analyses revealed similar findings for the major species of bacteria isolated from the tea, including *Streptococcus, Neisseria, Haemophilus, Gemella, Cutibacterium, Rothia, Veillonella, Actinomyces, Prevotella, Fusobacterium, Capnocytophaga*, and *Porphyromonas* (Table 2). 

Furthermore, the culture and the metagenomic analyses showed that *Streptococcus* species were the most commonly detected bacteria at the mouth of bottles 24 h after drinking (Table 1 and Table 2), and *Delftia, Neisseria, Haemophilus, Rothia, Sphingomonas*, and *Ralstonia* were also detected by the metagenomic analysis (Table 2).

In the remaining tea immediately after drinking (Table 1), *Streptococcus* (65.7%) and *Actinomyces* (14.4%) were predominant, followed by *Veillonella* (6.0%), *Prevotella* (3.5%), *Schaalia* (3.0%), *Gemella* (1.5%), and *Rothia* (1.0%). In contrast, *Streptococcus* (85.9%) and *Cutibacterium* (12.7%) were the major species of isolated bacteria from the remaining tea 24 h after drinking (Table 1). The figure format of Table 1 and Table 2 can be found in Supplementary Materials.

The bacterial contaminations of foods [5,6] and drinks in PET bottles after drinking [1,2] have been reported, although most of these studies focused mainly on *E. coli* and other general (water resource-related) bacteria. However, no information is available on the impact of bacteria at the mouth of plastic bottles after drinking directly from the bottles, as it is generally recommended that any remaining beverage should be discarded. In the present study, to obtain scientific evidence of the bacterial contamination of plastic-bottled drinks, analyses of the bacteria at the mouth of plastic bottles after drinking directly from the bottles and in the remaining tea in the plastic bottles were performed under suitable culture conditions for oral microbiota, i.e., an appropriate culture medium, incubation temperature, and gaseous environment, since the obtained bacteria are assumed to originate from the oral cavity. The culture data were compared to and confirmed by the data from the metagenomic analysis in the present study (Table 2). In addition, analyses of the remaining tea after storage at 37 °C for 24 h were performed under culture conditions suitable for the growth of oral microbiota, which are similar to the storage conditions of remaining bottled drinks in the summertime or in hot weather. The findings of the present study demonstrated that oral bacteria were present at the mouth of the bottles, as well as in the remaining tea after drinking, and that *Streptococcus* species were predominant bacteria in the tea 24 h after drinking. Although the unsweetened tea used in the present study contains little catechins and caffeine, it has been reported that Japanese green tea containing substantial amounts of catechins inhibited the growth of bacteria after storage at 23.4–25.6 °C for 8 h [7]. 

*Streptococcus, Actinomyces, Veillonella, Cutibacterium, Prevotella, Schaalia, Gemella, Rothia*, and *Neisseria* were the predominant bacteria immediately after drinking, and among them, the reason why *Streptococcus* species can specifically survive 24 h after drinking was unknown.

Analyses of the microbiota profiles of a sport-drink (sweetened, pH < 4.0) and orange juice (pH < 4.0) are currently in progress in our laboratory. To date, we have found predominant bacteria, such as *Streptococcus, Actinomyces, Rothia, Prevotella, Neisseria, Veillonella, Schaalia,* and *Gemella* from a sport-drink; and *Streptococcus, Actinomyces, Gemella, Veillonella,* and *Prevotella* from orange juice) similar to those found in the present study (Table 1) (Kawachi M et al., unpublished data).

In summary, approximately 10,000 cells/mL of oral bacteria, including *Streptococcus, Actinomyces, Schaalia, Gemella, Cutibacterium*, and *Veillonella*, were found at the mouth of plastic bottles and in the remaining tea in the bottles immediately after drinking, and the composition of the bacteria was confirmed by metagenomic analysis. The bacterial levels increased 100-fold after storage at 37 °C for 24 h, and *Streptococcus* species were the predominant bacteria isolated from the mouth of the plastic bottles and in the remaining tea in the bottles, suggesting that any remaining tea in plastic bottles should be discarded or preserved in a refrigerator after drinking directly from the bottle.

## Figures and Tables

**Table 1:** Bacteria isolated from plastic bottles of tea immediately after drinking and 24 h after drinking.

	Tea Drink of Plastic Bottles				At the Mouth of Plastic Bottles			
	Immediately after Drinking (n = 10)		24 h after Drinking (n = 12)		Immediately after Drinking (n = 11)		24 h after Drinking (n = 8)	
Mean ± SD (CFU/mL)	(0.8 ± 1.6) × 10^4^		(2.5 ± 2.6) × 10^6^		(1.8 ± 1.7) × 10^4^		(1.4 ± 1.5) × 10^4^	
Total	201	(%)	213	(%)	309	(%)	141	(%)
Anaerobes	28	(13.9)	27	(12.7)	41	(13.3)	0	(0)
*Cutibacterium*	9	(4.5)	27	(12.7)	15	(4.9)		
*C. acnes*	9		27		15			
*Veillonella*	12	(6.0)			11	(3.6)		
*V. parvula/tobetsuensis*	12				11			
*Prevotella*	7	(3.5)			5	(1.6)		
*P. loescheii*	1				4			
*P. melaninogenica*	6				1			
*Fusobacterium*					3	(1.0)		
*F. periodonticum*					3			
*Porphyromonas*					3	(1.0)		
*P. pasteri/bronchialis*					3			
*Selenomonas*					1	(0.3)		
*S. noxia*					1			
*Leptotrichia*					1	(0.3)		
*L.* sp					1			
*Solobacterium*					1	(0.3)		
*S. moorei*					1			
Facultative anaerobes	162	(80.6)	178	(83.6)	259	(83.8)	137	(97.2)
*Streptococcus*	132	(65.7)	183	(85.9)	185	(59.9)	136	(96.5)
*S. mitis/oralis/infantis*	102		172		129		120	
*S. parasanguinis*	12		5		30		7	
*S. australis*	8		5		8		1	
*S. salivarius*	5				13			
*S. cristatus*	5		1		5		8	
*Actinomyces*	29	(14.4)			15	(4.9)		
*A. oris/naeslundi*	28				15			
*A. johnsonii*	1							
*Schaalia*	6	(3.0)			17	(5.5)		
*S. odontolytica*	6				17			
*Gemella*	3	(1.5)			17	(5.5)		
*G. sanguinis*					10			
*G. haemolyssans/parahaemolyssans*	3				7			
*Staphylococcus*			2	(0.9)	7	(2.3)	1	(0.7)
*S. epidermidis*			2		5		1	
*S. warneri/pasteuri*					2			
*Neisseria*	1	(0.5)	1	(0.5)	5	(1.6)		
*N. mucosa*					2			
*N. perflava*	1		1		2			
*N. oralis*					1			
*Rothia*	2	(1.0)			8	(2.6)		
*R. aeria*	1				3			
*R. mucilaginosa*					5			
*R. dentocariosa*	1							
*Haemophilus*					2	(0.6)		
*H. parainfluenzae*					2			
*Capnocytophaga*					1	(0.3)		
*C. gingivalis*					1			
*Abiotrophia*					1	(0.3)		
*A. defectiva*					1			
Unknown	8	(4.0)	2	(0.9)	4	(1.3)	2	(1.4)
Lost	3	(1.5)	6	(2.8)	5	(1.6)	2	(1.4)

**Table 2:** Culture and metagenomic analyses of the bacterial compositions (percentages) at the mouth of plastic bottles immediately after and 24 h after drinking.

	Immediately after Drinking		24 h after Drinking	
	Culture (n = 11)	Metagenomic (n = 3)	Culture (n = 8)	Metagenomic (n = 3)
*Streptococcus*	59.870	10.711	96.454	46.586
*Delftia*		34.506		17.907
*Neisseria*	1.618	24.245		14.608
*Haemophilus*	0.647	15.658		8.926
*Schaalia*	5.502			
*Gemella*	5.502	0.381		0.320
*Cutibacterium*	4.854	0.041		0.308
*Rothia*	2.589	4.170		4.509
*Veillonella*	3.560	1.130		0.651
*Actinomyces*	4.854	0.406		0.351
*Lautropia*		3.083		0.328
*Sphingomonas*		1.745		2.374
*Prevotella*	1.618	0.442		0.352
*Ralstonia*		1.436		1.090
*Fusobacterium*	0.971	0.461		0.214
*Capnocytophaga*	0.324	0.028		
*Agrobacterium*		0.394		0.185
*Porphyromonas*	0.971	0.060		0.114
*Abiotrophia*	0.324			
*Bergeyella*		0.344		0.142
*Burkholderia*				0.320
*Corynebacterium*		0.289		0.074
*Ochrobactrum*		0.208		0.123
TM7 [G-1]		0.031		0.142
*Granulicatella*		0.010		0.126
*Bradyrhizobium*		0.099		0.098
*Bosea*		0.026		0.076
*Bacillus*				0.072
*Peptostreptococcus*		0.019		
*Alloprevotella*		0.019		
*Ruminococcaceae* [G-1]		0.012		
*Aggregatibacter*		0.012		
*Tannerella*		0.011		
*Campylobacter*		0.009		0.002
*Escherichia*		0.009		
*Catonella*		0.005		
*Cardiobacterium*		0.003		
TM7 [G-3]				0.002
*Fretibacterium*				0.002
*Selenomonas*		0.001		
*Peptostreptococcaceae* [XI] [G-1]				0.001
Unknown	1.294		1.418	
Lost	1.618		1.418	

## Data Availability

The data presented in this study are available on request from the corresponding author. The data are not publicly available due to privacy reasons for the subjects in this study.

## Supplementary Materials

The following are available online at https://www.mdpi.com/article/10.3390/dj9060058/s1, Figure S1: Data of Table 1 presented as a figure format, Figure S2: Data of Table 2 presented as a figure format.

## References

[1] Ohnishi T., Goto K., Kanda T., Kanazawa Y., Ozawa K., Sugiyama K., Watanabe M., Konuma H., Hara-Kudo Y. (2013). Microbial contamination associated with consumption and the growth in plastic bottled beverage. J. Environ. Sci. Health A Tox. Hazard. Subst. Environ. Eng..

[2] Watanabe M., Ohnishi T., Araki E., Kanda T., Tomita A., Ozawa K., Goto K., Sugiyama K., Konuma H., Hara-Kudo Y. (2014). Characteristics of bacterial and fungal growth in plastic bottled beverages under a consuming condition model. J. Environ. Sci. Health A Tox. Hazard. Subst. Environ. Eng..

[3] Ishida N., Sato T., Hoshikawa Y., Tanda N., Sasaki K., Kondo T., Takahashi N. (2015). Microbiota profiling of bronchial fluids of patients with pulmonary carcinoma. J. Oral. Biosci..

[4] Sato T., Yamaki K., Ishida N., Hashimoto K., Takeuchi Y., Shoji M., Sato E., Matsuyama J., Shimauchi H., Takahashi N. (2012). Cultivable anaerobic microbiota of infected root canals. Int. J. Dent..

[5] Kim M.J., Kim S.A., Kang Y.S., Hwang I.G., Rhee M.S. (2013). Microbial diversity and prevalence of foodborne pathogens in cheap and junk foods consumed by primary schoolchildren. Lett. Appl. Microbiol..

[6] Kim S.A., Oh S.W., Lee Y.M., Imm J.Y., Hwang I.G., Kang D.H., Rhee M.S. (2011). Microbial contamination of food products consumed by infants and babies in Korea. Lett. Appl. Microbiol..

[7] Morioka I., Uenaka A., Tanigawa A., Matsumoto Y. (2018). Microbial growth in unfinished beverages in plastic bottles and the awareness of nursing students in a university about microbial contamination. Jpn. J. Hyg..

