# Supplementary material for: Profiling of Microbiota at the Mouth of Bottles and in Remaining Tea after Drinking Directly from Plastic Bottles of Tea
Source: Dent J (Basel). 2021 May 21;9(6):58. doi: 10.3390/dj9060058 (PMC8224315; doi:10.3390/dj9060058)
Supplement: Supplementary file 1 [file dentistry-09-00058-s001.zip › dentistry-1216253-supplementary.pptx]

## Slide 1
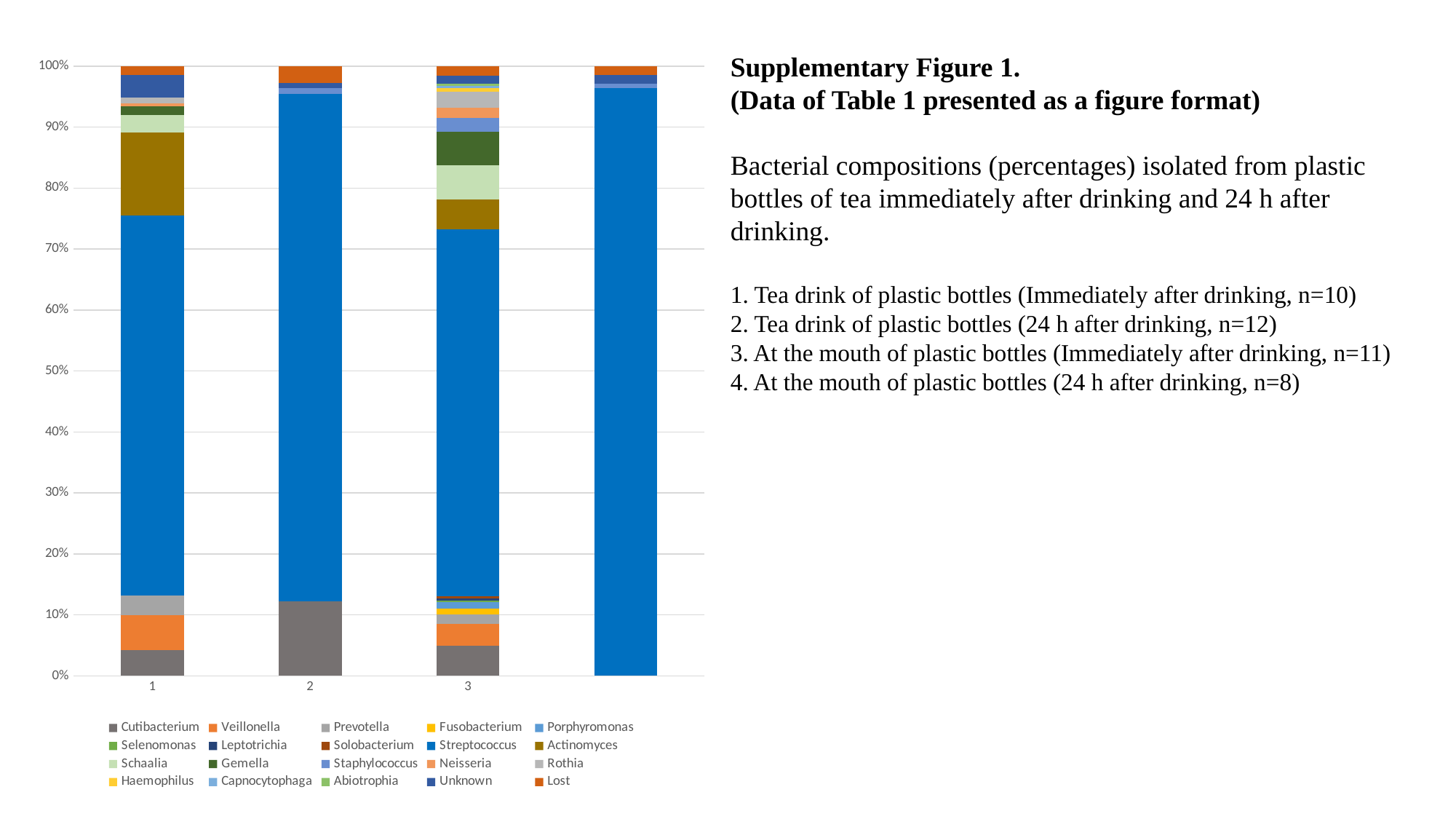

### Chart
| Category | Cutibacterium | Veillonella | Prevotella | Fusobacterium | Porphyromonas | Selenomonas | Leptotrichia | Solobacterium | Streptococcus | Actinomyces | Schaalia | Gemella | Staphylococcus | Neisseria | Rothia | Haemophilus | Capnocytophaga | Abiotrophia | Unknown | Lost |
|---|---|---|---|---|---|---|---|---|---|---|---|---|---|---|---|---|---|---|---|---|Supplementary Figure 1.
(Data of Table 1 presented as a figure format)
Bacterial compositions (percentages) isolated from plastic bottles of tea immediately after drinking and 24 h after drinking.
1. Tea drink of plastic bottles (Immediately after drinking, n=10)
2. Tea drink of plastic bottles (24 h after drinking, n=12)
3. At the mouth of plastic bottles (Immediately after drinking, n=11)
4. At the mouth of plastic bottles (24 h after drinking, n=8)

## Slide 2
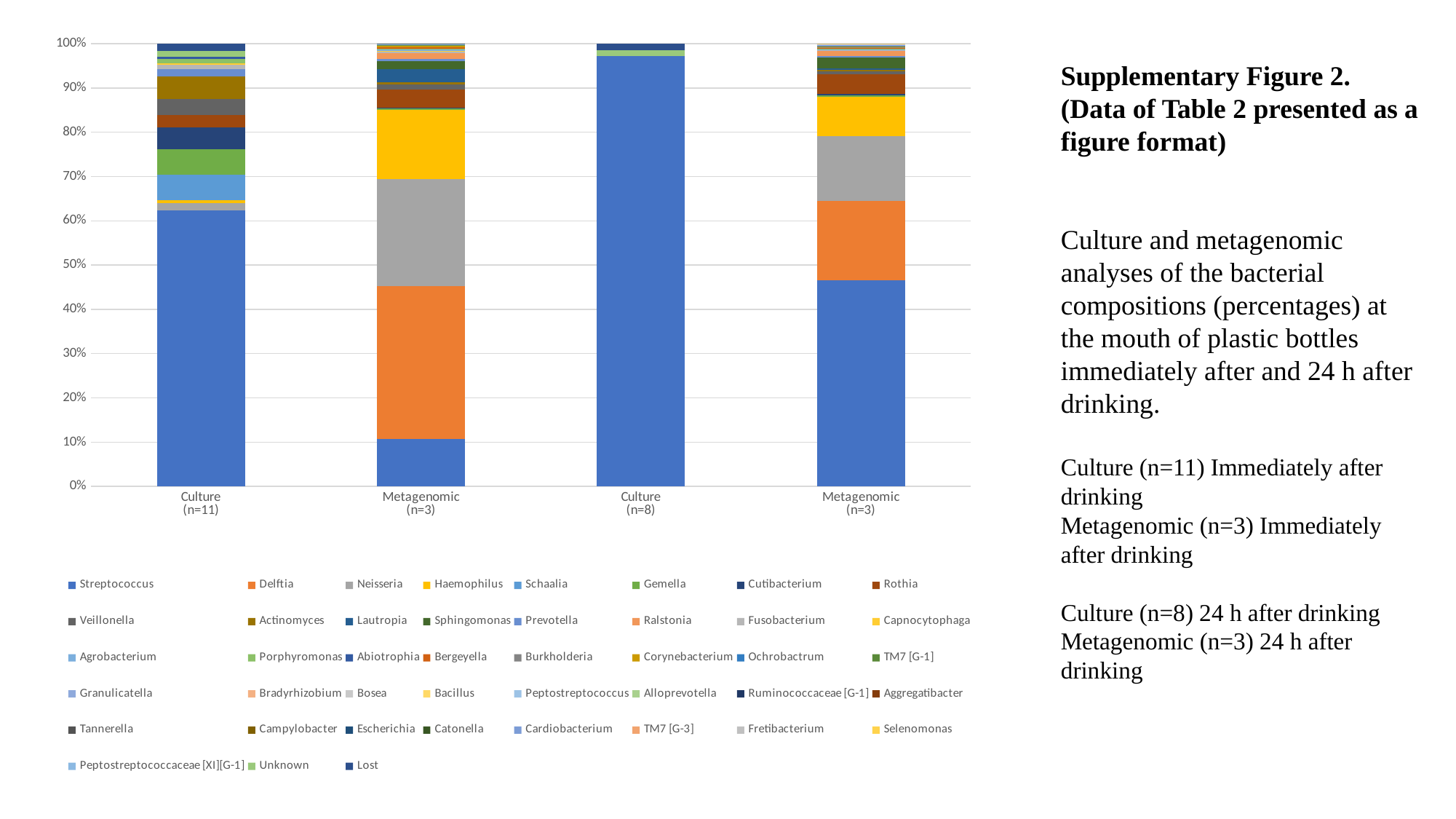

### Chart
| Category | Streptococcus | Delftia | Neisseria | Haemophilus | Schaalia | Gemella | Cutibacterium | Rothia | Veillonella | Actinomyces | Lautropia | Sphingomonas | Prevotella | Ralstonia | Fusobacterium | Capnocytophaga | Agrobacterium | Porphyromonas | Abiotrophia | Bergeyella | Burkholderia | Corynebacterium | Ochrobactrum | TM7 [G-1] | Granulicatella | Bradyrhizobium | Bosea | Bacillus | Peptostreptococcus | Alloprevotella | Ruminococcaceae [G-1] | Aggregatibacter | Tannerella | Campylobacter | Escherichia | Catonella | Cardiobacterium | TM7 [G-3] | Fretibacterium | Selenomonas | Peptostreptococcaceae [XI][G-1] | Unknown | Lost |
|---|---|---|---|---|---|---|---|---|---|---|---|---|---|---|---|---|---|---|---|---|---|---|---|---|---|---|---|---|---|---|---|---|---|---|---|---|---|---|---|---|---|---|---|
| Culture
(n=11) | 59.87 | None | 1.618 | 0.647 | 5.502 | 5.502 | 4.854 | 2.589 | 3.56 | 4.854 | None | None | 1.618 | None | 0.971 | 0.324 | None | 0.971 | 0.324 | None | None | None | None | None | None | None | None | None | None | None | None | None | None | 0.0 | None | None | None | None | None | None | None | 1.294 | 1.618 |
| Metagenomic
(n=3) | 10.711 | 34.506 | 24.245 | 15.658 | None | 0.381 | 0.041 | 4.17 | 1.13 | 0.406 | 3.083 | 1.745 | 0.442 | 1.436 | 0.461 | 0.028 | 0.394 | 0.06 | None | 0.344 | None | 0.289 | 0.208 | 0.031 | 0.01 | 0.099 | 0.026 | None | 0.019 | 0.019 | 0.012 | 0.012 | 0.011 | 0.009 | 0.009 | 0.005 | 0.003 | None | None | 0.001 | None | None | None |
| Culture
(n=8) | 96.454 | None | None | None | None | None | None | None | None | None | None | None | None | None | None | None | None | None | None | None | None | None | None | None | None | None | None | None | None | None | None | None | None | None | None | None | None | None | None | None | None | 1.418 | 1.418 |
| Metagenomic
(n=3) | 46.586 | 17.907 | 14.608 | 8.926 | None | 0.32 | 0.308 | 4.509 | 0.651 | 0.351 | 0.328 | 2.374 | 0.352 | 1.09 | 0.214 | None | 0.185 | 0.114 | None | 0.142 | 0.32 | 0.074 | 0.123 | 0.142 | 0.126 | 0.098 | 0.076 | 0.072 | None | None | None | None | None | 0.002 | None | None | None | 0.002 | 0.002 | None | 0.001 | None | None |Supplementary Figure 2.
(Data of Table 2 presented as a figure format)
Culture and metagenomic analyses of the bacterial compositions (percentages) at the mouth of plastic bottles immediately after and 24 h after drinking.
Culture (n=11) Immediately after drinking
Metagenomic (n=3) Immediately after drinking
Culture (n=8) 24 h after drinking
Metagenomic (n=3) 24 h after drinking
